# Effects of a ROCK Inhibitor on Retinal Ganglion Cells In Vivo and In Vitro

**DOI:** 10.3390/jcm14155344

**Published:** 2025-07-29

**Authors:** Wanjing Chen, Yoko Iizuka, Fumihiko Mabuchi, Kenji Kashiwagi

**Affiliations:** Department of Ophthalmology, School of Medicine, University of Yamanashi, Yamanashi 409-3898, Japan; g22ddm12@yamanashi.ac.jp (W.C.); showayk@hotmail.com (Y.I.); fmabuchi@icloud.com (F.M.)

**Keywords:** ROCK inhibitor, retinal ganglion cell, glaucoma

## Abstract

**Objective**: To investigate the neuroprotective effects of a Rho-associated kinase (ROCK) inhibitor on retinal ganglion cells (RGCs) in vitro and in vivo. **Methods**: For in vivo studies, a unilateral optic nerve crush mouse model was established. Then, 100 mM Y-27632 (a ROCK inhibitor) or saline was applied to the experimental eyes once a day for 14 days. The effects of the ROCK inhibitor were evaluated by counting the surviving RGCs in the enucleated flat retina tissues and measuring the inner retinal thickness using optical coherence tomography (OCT), the amplitude of the electroretinogram (ERG), and the change in intraocular pressure (IOP). For the in vitro study, RGCs were isolated from five-day-old mice using a modified immunopanning method with magnetic beads. The isolated RGCs were incubated for 72 h with various concentrations of Y-27632, after which TUNEL assays were performed to determine the number of surviving RGCs. **Results**: Y-27632 has neuroprotective effects, as it significantly increased the number of surviving RGCs by approximately 6.3%. OCT and ERG data also revealed that Y-27632 induced neuroprotective effects in vivo; furthermore, Y-27632 reduced IOP by approximately 18.3%. The in vitro study revealed the dose-dependent neuroprotective effects of Y-27632, with the highest dose of Y-27632 (1000 nM) increasing the RGC survival rate after 72 h of incubation compared with that of the control. **Conclusions**: The ROCK inhibitor Y-27632 may exert some neuroprotective effects on RGCs when it is used as an eye drop through an IOP-independent mechanism.

## 1. Introduction

The eye is a vital sensory organ that is responsible for approximately 80% of the external information that humans perceive. However, once ocular diseases, including glaucoma, damage visual function, recovery is often difficult. Therefore, preventing the progression of optic neuropathy is the goal of current treatments [1,2]. In recent years, Rho-associated kinase (ROCK) inhibitors, a novel class of therapeutic agents in ophthalmology, have been developed as antiglaucoma eye drops [3,4,5,6]. Netarsudil was approved for use in glaucoma treatment in the USA in late 2017 [7], and fasudil was approved for use in Japan in 1995 [4,8,9].

ROCK inhibitors for ophthalmological applications have been reported to have several effects [10], such as controlling aqueous outflow, protecting trabecular meshwork cells from oxidative stress [11], improving blood flow to the optic nerve [12,13,14], increasing ganglion cell survival, providing neuroprotection [15,16], repairing the corneal endothelium [17,18], and inhibiting bleb scarring during glaucoma surgery [19].

The ROCK inhibitor trans-4-[(1R)-aminoethyl]-N-(4-pyridinyl)cylohexanecarboxamide dihydrochloride (Y-27632) has been shown in recent animal studies to promote corneal wound healing [17,20,21,22], mitigate hypoxia and oxidative-induced injury to retinal Müller cells [23], and reduce scarring during glaucoma filtering surgery [17]. Moreover, Y-27632 has also been demonstrated to influence intraocular pressure (IOP) by adjusting aqueous humor drainage [24,25].

Previous studies have indicated that Y-27632 may protect retinal ganglion cells (RGCs) via an IOP-independent mechanism in addition to its ability to lower the IOP directly [26]. However, it remains unclear whether Y-27632 offers neuroprotection to RGCs in ways other than reducing IOP. Therefore, the aim of this study was to investigate the neuroprotective effects of Y-27632 on RGCs in vivo in an optic nerve crush (NC) model and in vitro.

## 2. Materials and Methods

### 2.1. Animals

C57BL/6J mice (wild-type) and B6. Cg-Tg(Thy1-CFP)23Jrs/J mice (referred to as Thy1-CFP-mice) were used experimentally in accordance with the ARVO Statement for the Use of Animals in Ophthalmic and Vision Research, the ‘Guiding Principles in the Care and Use of Animals in the Field of Physiologic Sciences’ published by the Physiologic Society of Japan, and with the previous approval of the Animal Care Committee of Yamanashi University. The experiments were designed to minimize the number of animals euthanized and their suffering.

### 2.2. ROCK Inhibitor

The ROCK inhibitor Y-27632 was purchased from LC Laboratories (Y-5301) (LC Laboratories, Woburn, MA, USA) and was prepared in deionized water (DIW). The Y-27632 solution was sterilized by filtration using a syringe filter prior to application.

### 2.3. Optic NC Injury and Eye Drop Instillation

A unilateral optic NC model was established in adult Thy1-CFP-mice (10~20 weeks old) according to a previous method [27]. In each mouse, one eye was subjected to NC, while the contralateral eye served as the control. This within-subject paired design minimized inter-animal variability and enhanced statistical power. Anesthesia was induced via intraperitoneal injection of ketamine (1.6 mL/kg) and xylazine (0.8 mL/kg). A small cut was made in the conjunctiva inferior to the globe and continued around the eye. The optic nerve was then crushed 1 mm behind the eyeball with Dumont #N7 cross-action forceps (RS-5027; Roboz Surgical Instrument Co., Inc., Gaithersburg, MD, USA) by applying pressure for 15 s while avoiding injury to the ophthalmic artery. After surgery, 5 µL of 100 mM Y-27632 or saline was applied to the experimental eyes once a day for 14 days. As the use of a large number of animals in in vivo experiments is challenging, preliminary studies were conducted to determine the optimal concentration of Y-27632. Initial in vivo assessments of 1 mM and 10 mM concentrations failed to demonstrate appreciable neuroprotective effects on RGC survival under our experimental conditions. Based on these findings, 100 mM was selected as the lowest concentration that consistently yielded a quantifiable neuroprotective benefit. Therefore, the manuscript focuses on the results obtained with 100 mM.

### 2.4. IOP Measurements

The IOP was measured using a TonoLab tonometer (iCare, Vantaa, Finland) after the mice were deeply anesthetized. The IOP of each eye was measured three times at one-minute intervals on day 0 before injury and day 14 after surgery.

### 2.5. Optic Fundus Imaging

The retinal fundus was observed with an SMZ1500 stereoscopic zoom microscope (SMZ1500, Nikon, Tokyo, Japan) after 14 days of eye drop treatment. Five microliters of Mydrin-P ophthalmic solution (Santen Pharmaceutical Co., Ltd., Osaka, Japan) was applied topically after the mice were deeply anesthetized to induce pupil mydriasis, and then the cornea was covered with 0.1% Hyalein ophthalmic solution (Santen Pharmaceutical) to prevent dryness. A fundoscopy lens (V90C; Volk Optical, Inc., Mentor, OH, USA) was used to enlarge the fundus field of view. Retinal images were captured using an iPhone 12 (Apple Inc., Cupertino, CA, USA) fitted with an I-NTER lens (MR-6i; Micronet Inc., Saitama, Japan). To ensure sufficient image quality, lighting conditions were standardized, and a consistent distance and angle between the lens and the ocular surface were maintained throughout image acquisition.

### 2.6. Retinal Electrophysiology/Electroretinography

A scotopic electroretinogram (ERG) was recorded on day 15 according to the instructions. The mice were dark-adapted overnight, and all the experimental procedures were carried out under dim red light. After anesthetization as described above, five microliters of Mydrin-M ophthalmic solution (Santen Pharmaceutical Co., Ltd., Osaka, Japan) was applied to each eye to dilate the pupil. The electrodes were moistened with SCOPISOL SOLUTION FOR EYE (Senju Pharmaceutical Co., Ltd., Osaka, Japan) and then placed against the corneal surface to measure electrical responses. The ERG responses elicited by flash intensities ranging from −5 log cd·s/m^2^ to −3 log cd·s/m^2^ were recorded to determine the scotopic threshold response (STR).

### 2.7. Flat-Mount Whole Retina Evaluation

Following 14 days of treatment with 100 mM Y-27632 or saline, a burn mark was made on the dorsal cornea with a burn needle to distinguish the retina position and direction. The eyes were then enucleated and incubated in 4% (*w*/*v*) paraformaldehyde in phosphate-buffered saline (PBS) at 4 °C overnight. The superior rectus muscle was identified according to the position of the burn mark and the choroid fissure. Flat retinal tissue was prepared according to previous methods [28,29,30]. In brief, the retinal tissue was first cut at the superior rectus muscle from the limbus toward the optic disc and then flat-mounted. Each field sampled from the optic nerve head to the edge of four retinal leaflets (280 × 373 µm^2^ per field, 16 fields per retina) was imaged with a BZ-X700 all-in-one fluorescence microscope (BZ-X700; KEYENCE, Osaka, Japan). Finally, the number of CFP-positive RGCs was determined.

**Figure 1:**
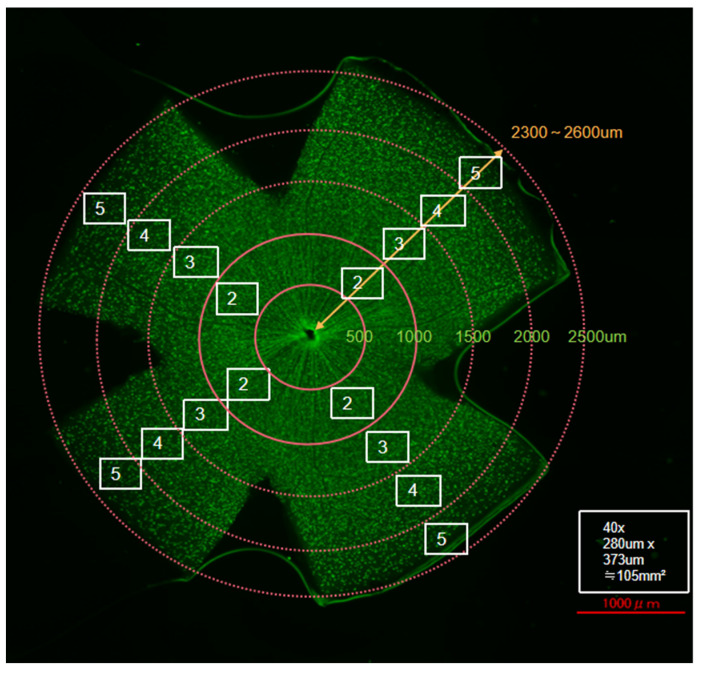
Schematic representation of retinal flat-mount quantification. Scale bar = 1000 µm. RGC counting regions were defined at specific distances from the optic nerve head in each quadrant as follows: Area 2, 500–1000 µm; Area 3, 1000–1500 µm; Area 4, 1500–2000 µm; and Area 5, 2000–2500 µm.

The surviving RGCs were directly counted using ImageJ software (ImageJ 1.53t; National Institutes of Health, Bethesda, MD, USA) according to the manufacturer’s instructions. Briefly, the RGCs in the four outer images of each retinal leaflet were counted via the image processing function of ImageJ. Particles with areas less than 20 µm^2^ were automatically removed and not counted. We subsequently calculated the number of surviving RGCs (or overall RGC density) in four regions (areas 2, 3, 4, and 5) separately, as shown in Figure 1.

### 2.8. Quantification of the Retinal Nerve Fiber Layer by Optical Coherence Tomography (OCT)

**Figure 2:**
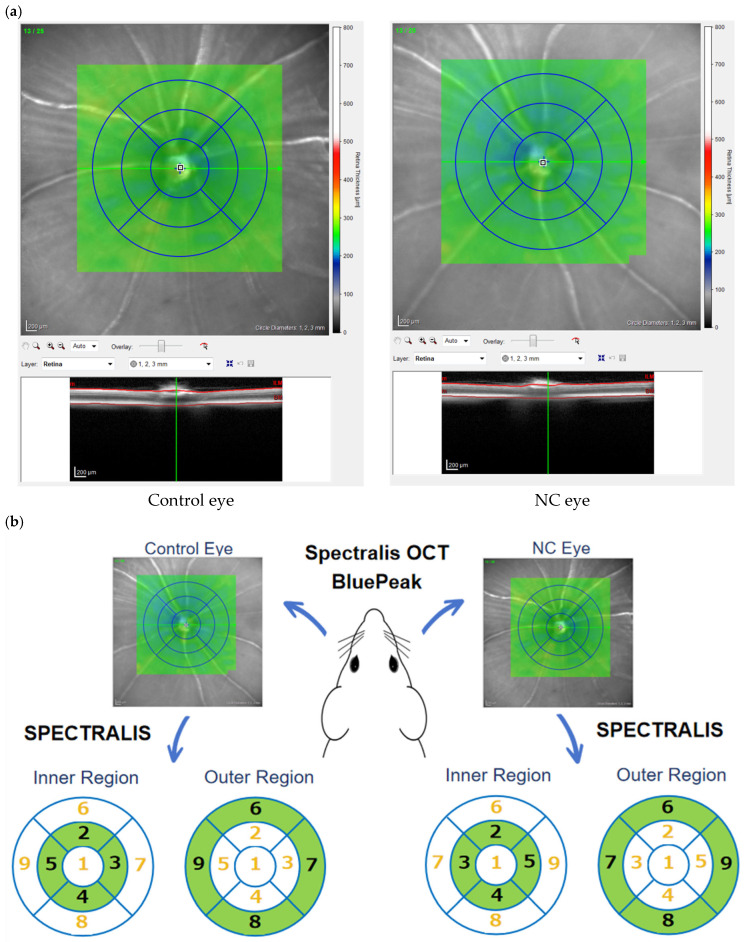
(**a**) Representative retinal images of a wild-type mouse after NC and 14 days of eye drop treatment. (**b**) OCT evaluation of retinal cross-sections. The four quadrants were divided into internal and external regions for analysis.

The thicknesses of the retina and ganglion cell complex (GCC), which includes the retinal nerve fiber layer (RNFL), retinal ganglion cell layer (RGCL), and inner plexiform layer (IPL), were evaluated by OCT (Spectralis OCT BluePeak; Heidelberg Engineering GmbH, Heidelberg, Germany) in NC model mice after 14 days of eye drop treatment. Briefly, five microliters of 0.4% SANDOL MY Ophthalmic Solution (ROHTO NITTEN Co., Ltd., Nagoya, Japan) were applied after full anesthesia for eye dilation. After the mice were positioned firmly on the measuring table, raster scan images centered on the optic disc of the retina with horizontal and vertical dimensions of 3.5 × 1.9 mm^2^ and depth to cover a 3.5 × 3.5 mm^2^ area of the retina were taken (Figure 2a). The dorsal, nasal, ventral and temporal areas in the inner region (500~1000 µm from the optic disc) were named areas 2, 3, 4, and 5, respectively, and the areas in the outer region (1000 µm~1500 µm from the optic disc) were named areas 6, 7, 8, and 9, respectively (Figure 2b). The thicknesses of the retinal layers in each area were measured with automated segmentation software (SPECTRALIS software V6.13-JP), and the automated segmentation was manually corrected in the case of clear mis-segmentation. Specifically, if the automated segmentation exhibited visible discontinuities, overlaps, or misaligned anatomical boundaries, a primary evaluator blinded to group allocation corrected the results. In uncertain cases, two to three observers independently reviewed the images and reached a consensus to ensure consistency and minimize bias.

### 2.9. RGC Isolation

RGCs were isolated via a modified magnetic bead immunopanning method [31,32,33]. Briefly, 5-day-old C57BL/6J mice were euthanized to obtain more than 10 eyes for each experimental group. After dissociation, the retinal cell suspension was incubated with a rabbit anti-mouse macrophage antibody (CLAD31240; Cedarlane, Burlington, ON, Canada) for 5 min and then for 30 min in 100 mm petri dishes coated with a goat anti-rabbit IgG antibody (Southernbiotech, Birmingham, AL, USA), a purified mouse anti-CD11b/c antibody (554859BD, Becton, Dickinson and Company, Franklin Lakes, NJ, USA), and a mouse anti-HNK-1/N-CAM monoclonal antibody (CD57) (C6680; Merck (Sigma-Aldrich), Darmstadt, Germany) to remove macrophages, microglia, endothelial cells, fibroblasts, and Thy1(+) amacrine cells from the samples. Nonadherent retinal cells were treated with a biotinylated anti-Thy-1.2 antibody (ab25285; Abcam, Cambridge, UK) for 60 min at 37 °C and subsequently incubated with antibiotin MicroBeads for 15 min at 4 °C. The magnetically labeled RGCs were collected using a magnetic separation unit (MACS; Miltenyi Biotec GmbH, Bergisch Gladbach, Germany), and the purified RGCs were cultured in Politi medium throughout the experiments [31,32].

### 2.10. Immunocytochemistry for RGC Identification and Purity Assessment

The isolated cells were fixed in 4% paraformaldehyde and permeabilized with 0.5% Triton X-100. After blocking, the cells were incubated overnight at 4 °C with primary antibodies against anti-Brn3a (MAB1585; Merck (Sigma-Aldrich), Darmstadt, Germany), anti-RBPMS (ab194213, Abcam, Cambridge, UK), and anti-Prox1 (925202; BioLegend, San Diego, CA, USA). Following washes, the samples were incubated with the following fluorescent secondary antibodies: goat anti-mouse IgG (ab150113; Abcam, Cambridge, UK) and goat anti-rabbit IgG (ab175471; Abcam, Cambridge, UK). Nuclei were counterstained with DAPI (sc-24941; Santa Cruz Biotechnology, Inc., Dallas, TX, USA). RGC purity was assessed by counting Brn3a(+), RBPMS(+), and Prox-1(−) cells using ImageJ (ImageJ 1.53t; National Institutes of Health, Bethesda, MD, USA).

### 2.11. TUNEL Assay

Dead and living cells were examined using an Apo-BrdU in situ DNA fragmentation assay kit (Cat #K401-60; BioVision, Milpitas, CA, USA) according to the manufacturer’s protocol. In brief, cultured cells were fixed with 4% (*w*/*v*) PFA, washed, incubated in 70% ethanol overnight at −20 °C, and then treated with DNA labeling solution for 60 min at 37 °C. After treatment with an anti-BrdU-FITC antibody for 30 min at room temperature in the dark, the cells were stained with propidium iodide/RNase for 30 min at room temperature. Dead and living cells were analyzed by fluorescence microscopy within 3 h of staining.

### 2.12. Statistical Analysis

Statistical analysis was performed using Microsoft Excel statistical software and SPSS Statistics (version 31.0, IBM Corporation, Armonk, NY, USA). The *t*-test and the Mann–Whitney U test were applied for comparisons between two groups to evaluate significant differences in flat-mount RGC survival rates. One-way ANOVA was performed to analyze changes in IOP. The data are presented as the means ± standard errors (with a minimum of n = 8 mice per condition in in vivo experiments, as mentioned in each result or figure), and the differences were considered significant at *p* < 0.05.

## 3. Results

### 3.1. Effects of Y-27632 on RGC Survival After NC

**Figure 3:**
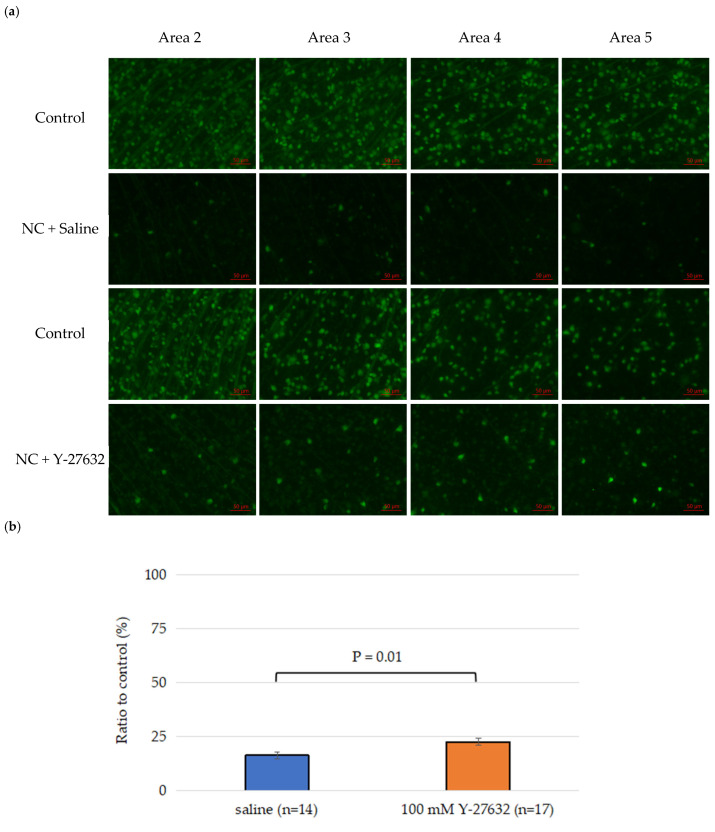

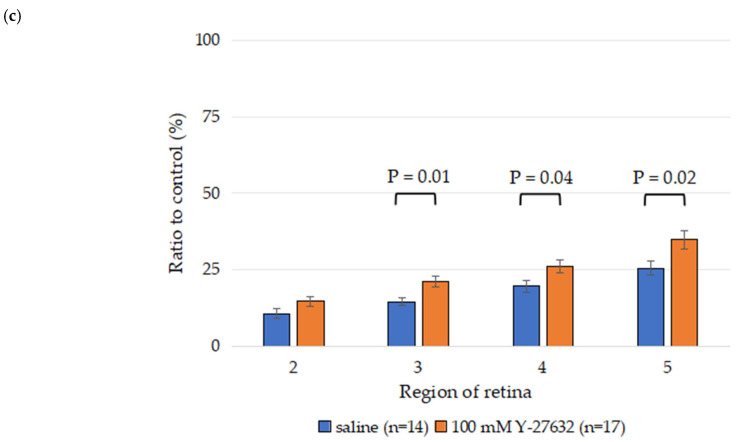
(**a**) Representative retinal flat-mount images from CFP mice showing RGC fluorescence signals in control, saline-treated, and Y-27632-treated eyes 14 days after NC. The images were taken from the same retinal leaflet in all three groups and correspond to sampling areas 2, 3, 4, and 5, as defined in Figure 1. Scale bar = 50 µm; (**b**) Total RGC survival rates; (**c**) Average RGC survival rates across the four sampling regions.

More than 80% of the RGCs had died in the model mice with optic nerve crush (NC) injury compared to the sham group after 14 days. Two weeks after NC injury, the total RGC survival rate in the saline treatment group was 16.3 ± 1.5%, whereas the 100 mM Y-27632 treatment group had a significantly greater RGC survival rate of 22.6 ± 1.7% (Figure 3b). The percentages of surviving RGCs in the saline group decreased to 10.6 ± 1.6%, 14.5 ± 1.3%, 19.6 ± 2.0%, and 25.5 ± 2.4% (n = 14) in areas 2, 3, 4, and 5, respectively. Moreover, the survival rates of the RGCs in the 100 mM Y-27632 group were 14.6 ± 1.6%, 21.0 ± 1.7%, 26.0 ± 2.2%, and 34.8 ± 3.0% (n = 17) in these same areas, respectively (Figure 3c). Compared with those in the saline group, significantly more RGCs survived in the Y-27632 treatment group (*p* < 0.05).

### 3.2. ERG Assessment of RGC Function

**Figure 4:**
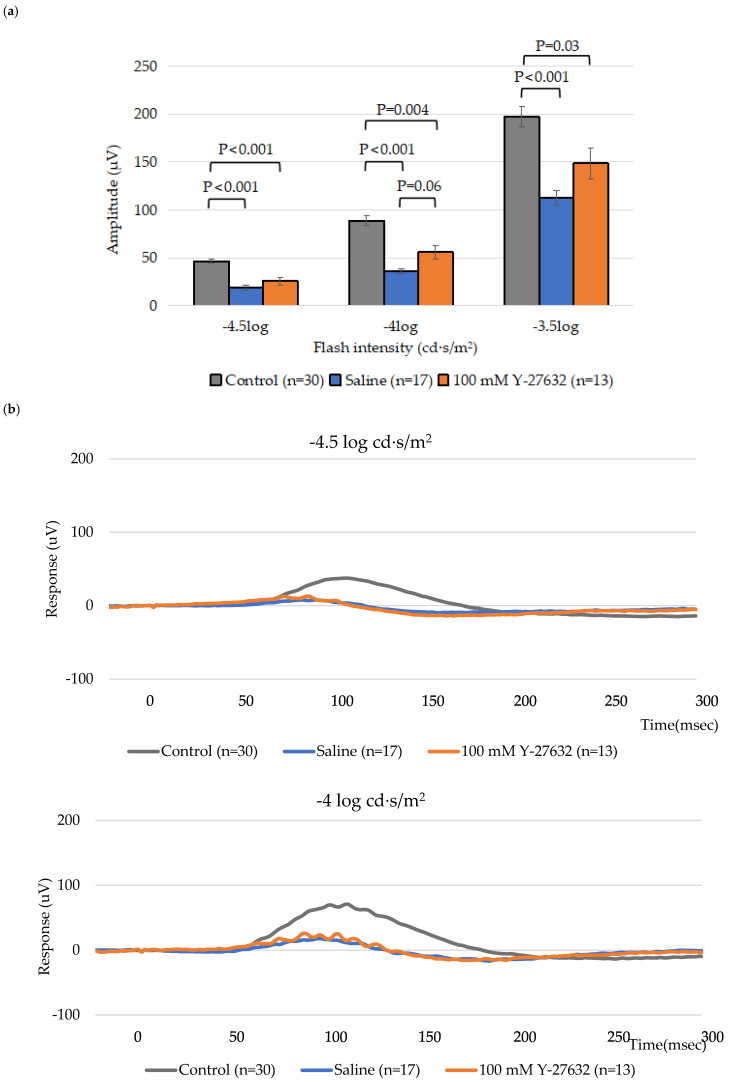

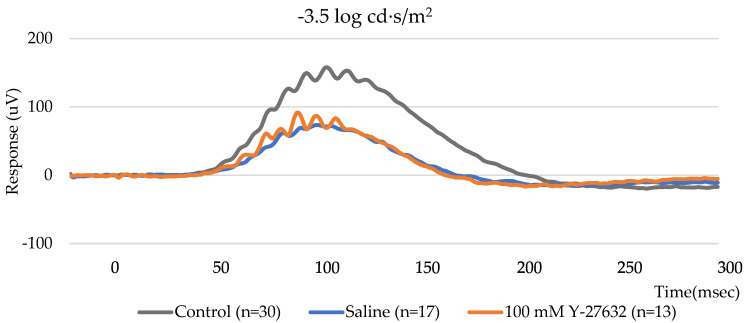
(**a**) Amplitude of the pSTR after 14 days of the eye drop experiment; (**b**) ERG traces recorded from NC mice treated with saline or 100 mM Y-27632 at a flash intensity of −4.5, −4, and −3.5 log cd·s/m^2^.

To study how the topical application of Y-27632 affects retinal and RGC function, scotopic ERGs were recorded on day 14. As expected, a significant reduction in the positive scotopic threshold response (pSTR) amplitude was detected following NC. The pSTR amplitudes of the control group were 46.7 ± 2.4 µV, 89.0 ± 4.8 µV, and 197.4 ± 10.7 µV (n = 30) at flash intensities of −4.5 log, −4 log and −3.5 log cd·s/m^2^, respectively, whereas the pSTR amplitudes of the saline group were 19.0 ± 2.8 µV, 36.0 ± 2.7 µV, and 112.8 ± 7.6 µV (n = 17). Notably, eyes treated with 100 mM Y-27632 showed higher pSTR amplitudes of 26.0 ± 4.1 µV, 56.1 ± 7.3 µV, and 148.7 ± 16.0 µV (n = 13), respectively, at the same flash intensities (Figure 4a). Thus, treatment with 100 mM Y-27632 increased the amplitudes that decreased in response to modeling and saline treatment.

To further evaluate the group differences across flash intensities, the average pSTR amplitudes were plotted as a function of stimulus intensity (Figure 4b). The Y-27632-treated eyes showed slightly higher pSTR amplitudes than the saline-treated eyes across all tested flash intensities. Although the differences were not pronounced, the trend suggests a modest improvement in RGC responsiveness following Y-27632 treatment.

### 3.3. Effects of NC and Y-27632 on RGCs Determined by Fundus Imaging

**Figure 5:**
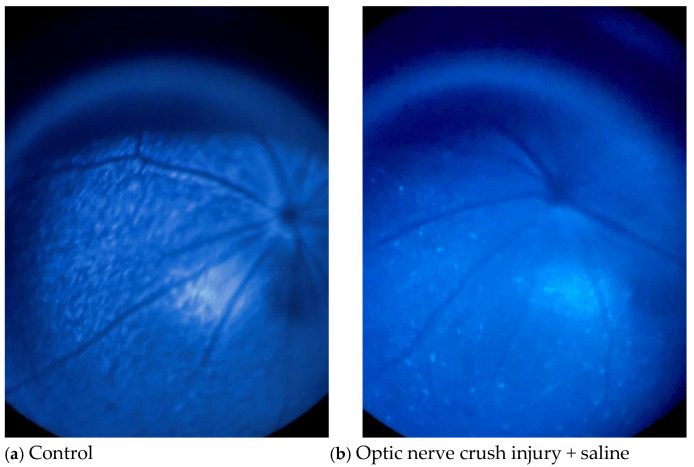

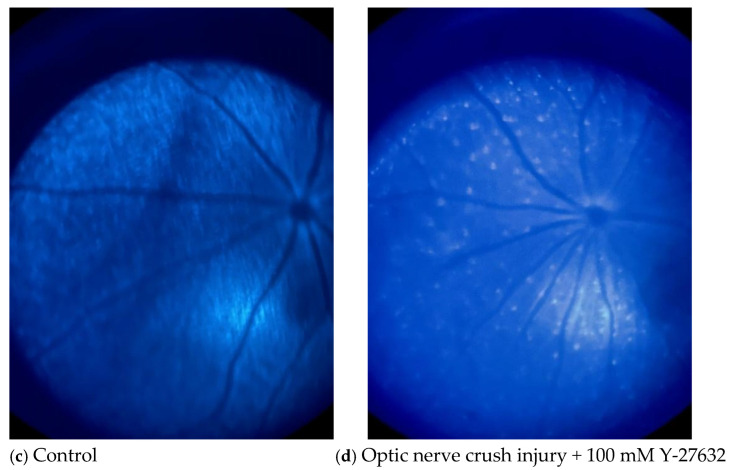
Representative mouse fundus images taken after two weeks of eye drop treatment. (**a**,**c**) Untreated control eyes; (**b**) saline-treated eyes after NC; (**d**) Y-27632-treated eyes after NC. All images were captured from the same retinal region under identical imaging conditions.

Figure 5 shows two pairs of fluorescence images of the mouse fundus taken on day 15 (the final day of treatment). Although the fluorescence intensities of both the saline- (Figure 5b) and Y-27632 (Figure 5d)-treated eyes were lower than those of the control eyes (Figure 5a,c), the fluorescence intensity of the retina and the number of CFP-positive RGCs were much greater in the Y-27632-treated eyes than in the saline-treated eyes.

### 3.4. OCT Quantification of the Retinal Layers

**Figure 6:**
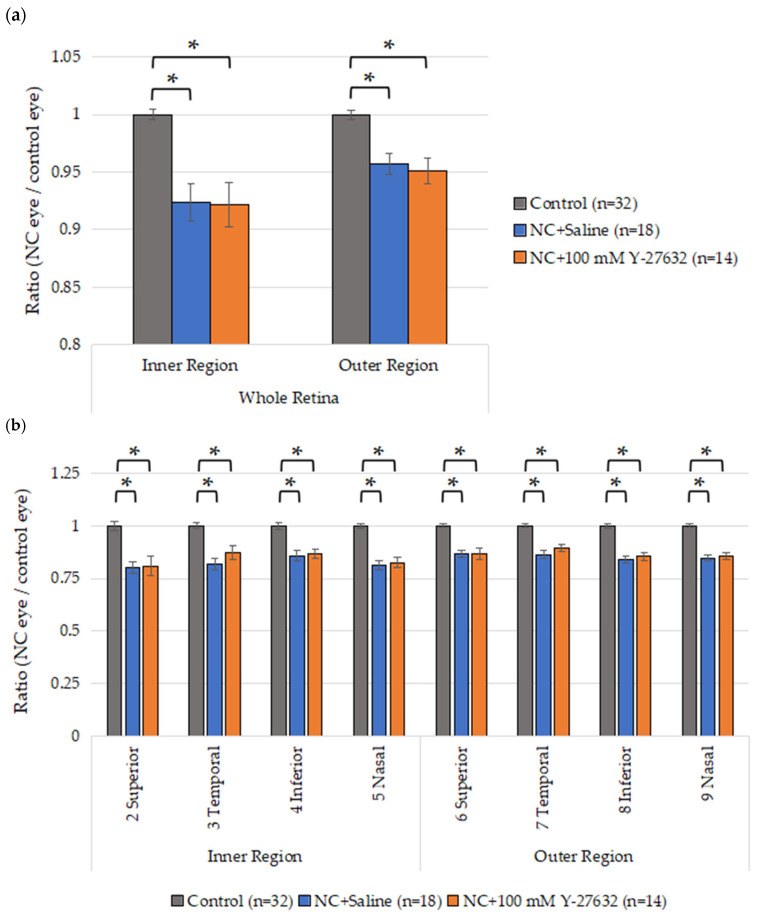
(**a**) Average retinal thickness after NC injury and two weeks of eye drop instillation; (**b**) Ratio of the GCC thickness in each area. * *p* < 0.001.

To evaluate the morphological changes in the retinas of the NC model mice induced by Y-27632, OCT was performed after two weeks of eye drop treatment. Fourteen days after NC injury, the average whole retina thickness in the inner region was 244.6 ± 1.6 µm, 225.9 ± 5.9 µm, and 225.5 ± 6.7 µm in the control group (n = 32), saline group (n = 18), and Y-27632 group (n = 14), respectively. Additionally, these averages were 252.6 ± 1.5 µm, 241.8 ± 3.3 µm, and 240.2 ± 4.0 µm in the outer regions of the control group (n = 32), saline group (n = 18), and Y-27632 group (n = 14), respectively. Compared with the control eyes, the experimental eyes presented a significant reduction in retinal thickness, but there was no difference between the two experimental groups (Figure 6a). However, the GCC thicknesses of the experimental eyes were also significantly reduced, and the ratio of the GCC thickness (determined as the GCC thickness of the experimental eye divided by that of the control eye) after two weeks of treatment with 100 mM Y-27632 tended to increase compared with that after saline treatment (Figure 6b).

### 3.5. Effects of Y-27632 on IOP

**Figure 7:**
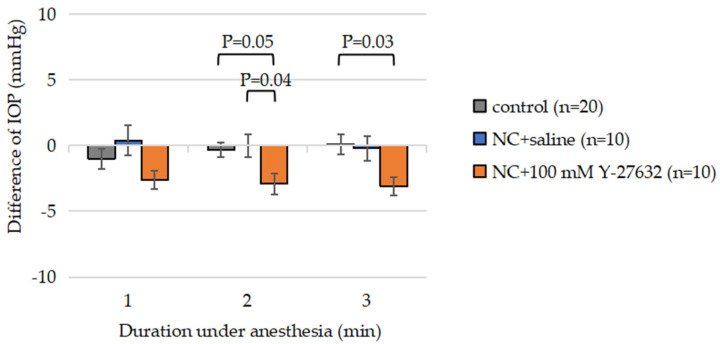
Average IOP before and after NC and treatment with 100 mM Y-27632 or saline. Compared with the control and saline-treated eyes, the Y-27632-treated eyes presented a significantly lower IOP.

To investigate the influence of Y-27632 on IOP following unilateral NC injury in adult mice, IOP was measured at two time points: day 0 (prior to surgery) and day 14 (post-injury). The average baseline IOP before injury was 15.7 mmHg. Compared with uninjured control eyes, saline-treated eyes showed no significant change in IOP. In contrast, eyes treated with 100 mM Y-27632 exhibited a consistent reduction in IOP, measured at one-minute intervals following deep anesthesia, with mean decreases of 2.6 ± 0.70 mmHg, 2.9 ± 0.81 mmHg, and 3.1 ± 0.67 mmHg at 1, 2, and 3 min, respectively (Figure 7).

These findings indicate that optic nerve injury alone did not significantly alter IOP, whereas topical administration of Y-27632 induces a modest yet reproducible reduction in IOP, corresponding to an average decrease of approximately 18.3% from baseline.

### 3.6. Confirmation and Purity Assessment of Primary Mouse RGCs

**Figure 8:**
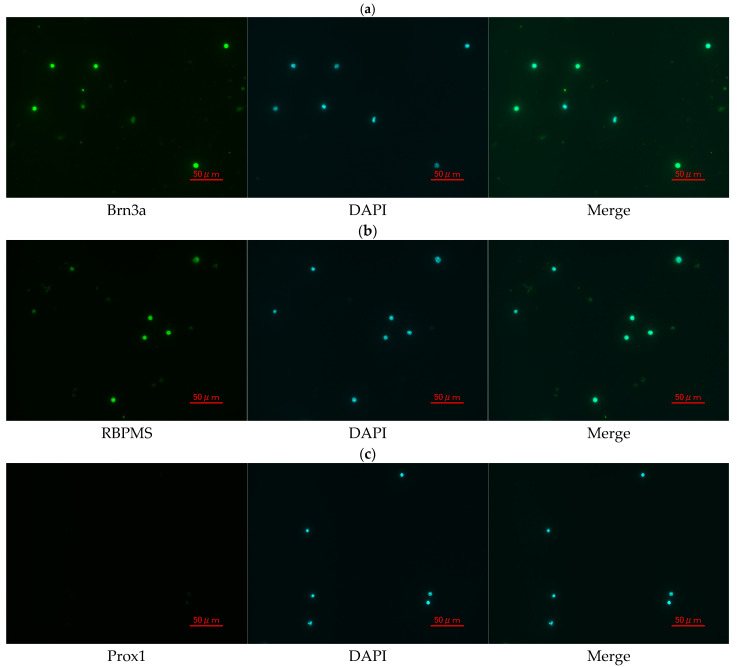
Immunofluorescence staining of primary mouse RGCs. (**a**) Brn3a (green), (**b**) RBPMS (green), and (**c**) Prox1 (green) staining, each shown alongside DAPI (blue) nuclear staining and merged images. Scale bar = 50 μm.

To evaluate the identity and purity of primary RGCs isolated from the mouse retina, immunofluorescence staining was performed using RGC-specific and non-RGC markers. As shown in Figure 8, approximately 95% of the isolated cells exhibited strong Brn3a immunoreactivity, confirming their identity as RGCs. More than 90% of the cells were also positive for RBPMS, another well-established marker of RGCs. Moreover, the non-RGC marker Prox1 was detected in fewer than 1% of the cells, indicating minimal contamination by other retinal cell types. These results demonstrate the high purity and successful isolation of RGCs from the mouse retina.

### 3.7. Effects of Y-27632 on Isolated RGCs

**Figure 9:**
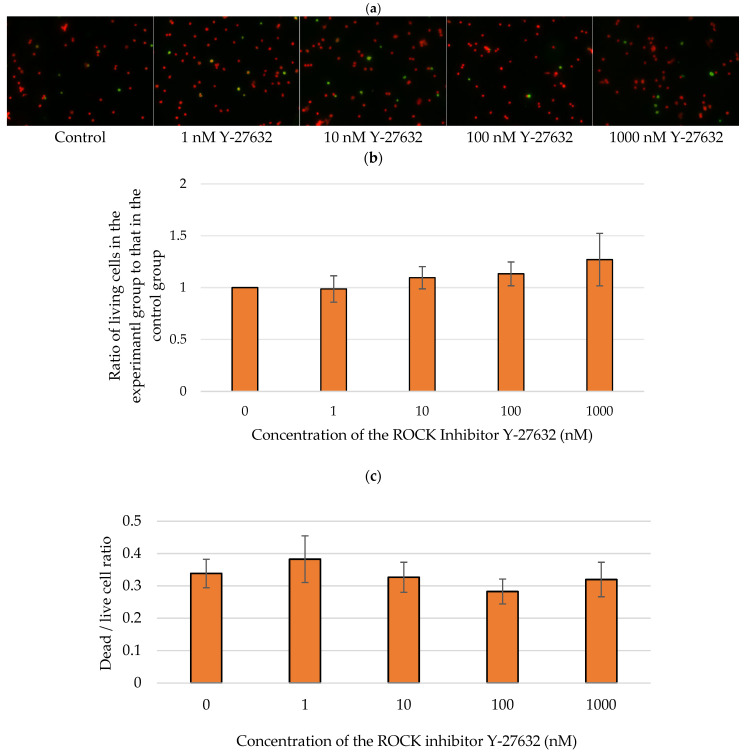
Y-27632 reduced isolated RGC death. (**a**) Images of TUNEL-positive (green) and PI-positive (red) RGCs after 72 h of incubation; (**b**) RGC survival rate after treatment with different concentrations of Y-27632; (**c**) Dead/living RGC ratio after 72 h of culture with various concentrations of Y-27632.

We further examined whether Y-27632 could directly affect RGC survival in vitro by treating RGCs with 0, 1, 10, 100, or 1000 nM Y-27632. Apoptotic RGCs were subsequently detected via TUNEL assays (Figure 9a). The average ratios of the number of living RGCs after 72 h of incubation with 1, 10, 100, and 1000 nM Y-27632 to the number of surviving control RGCs were 0.99 ± 0.13, 1.10 ± 0.11, 1.13 ± 0.11, and 1.27 ± 0.35, respectively (Figure 9b). Additionally, the average ratios of living to dead RGCs were 0.34 ± 0.04, 0.38 ± 0.07, 1.10 ± 0.11, 0.33 ± 0.05, 0.28 ± 0.04, and 0.32 ± 0.05 after culture with 0, 1, 10, 100, and 1000 nM Y-27632, respectively (Figure 9c). TUNEL assays revealed that the RGC survival rate increased with increasing Y-27632 concentration. Moreover, the dead/live RGC ratio tended to decrease as the concentration of Y-27632 increased. However, at the highest dose of Y-27632 (1000 nM), although the RGC survival rate was the highest, the dead/live cell ratio began to increase.

## 4. Discussion

The in vivo and in vitro results of the present study revealed that Y-27632 exerts a direct neuroprotective effect on RGCs. The in vivo optic NC model revealed that 100 mM Y-27632 can not only reduce IOP but also increase RGC survival after its application as an eye drop. Moreover, a similar neuroprotective effect of Y-27632 on RGCs was also found in vitro. Thus, Y-27632 may exert a direct neuroprotective effect on RGCs by mechanisms other than reducing IOP.

Y-27632 has previously been reported to affect the retina both in vivo and in vitro. Several prior studies using different animal models, retinal cells, or administration routes yielded results similar to those in our current report, indicating the neuroprotective effect of Y-27632 [26,34]. In addition, previous studies have suggested that Y-27632 alleviates optic neuropathy by regulating aqueous humor drainage [24,25].

In the present study, a unilateral optic NC mouse model was chosen, and although Y-27632 decreased the IOP, the mechanism of neuropathic disorder caused by optic NC injury may not be consistent with that of glaucoma. Unilateral optic NC model mice presented no significant difference in IOP between before NC injury and 14 days after NC injury. Moreover, Y-27632 lowered the IOP; however, it has not yet been proven that lowering the IOP alleviates damage to RGCs in a normal NC injury model. A previous study revealed that the effect of Y-27632 on RGCs may involve not only the regulation of IOP but also other mechanisms that do not involve IOP [35]. The neuroprotective effects of ROCK inhibitors have been noted [11,16], and Y-27632 has also been administered via intravitreal injection [26,36], anterior chamber [37] injection, or other methods in previous studies. However, from a clinical point of view, internal injection and oral administration are highly invasive for patients; thus, long-term compliance with these methods is difficult. In this in vivo study, the neuroprotective effect of Y-27632 was demonstrated after once-daily eye drop administration, a method that is easy for patients to accept, and the results also suggest that the neuroprotective effects of Y-27632 may be useful in the treatment of diseases other than glaucoma, such as traumatic neuropathy.

ROCK is ubiquitously expressed in most tissues and organs. The ROCK signaling pathway plays a role in a variety of cell functions, such as contraction, migration, cytokinesis, degeneration, and death. ROCK 1, which is highly expressed in nonneuronal tissues, and ROCK 2, which is highly expressed in the brain and muscles, are two such isoforms. Moreover, ROCK 1, ROCK 2, and several downstream effectors of the ROCK signaling pathway are expressed in the trabecular meshwork, which may affect IOP [11]. Our findings demonstrated that Y-27632 exerts direct neuroprotective effects on RGCs, and the available data also indicate that Y-27632 has the potential to protect RGCs independent of IOP when it is administered as an eye drop. At present, the expression of ROCK receptors in RGCs has not yet been fully elucidated, but the results of this study suggest that ROCK receptors may be expressed in RGCs. Although it is generally accepted that inhibiting the ROCK signaling pathway in RGCs can relieve RGC death, the latent mechanisms associated with these neuroprotective effects are still being investigated. It is difficult to distinguish which isoform ROCK inhibitors inhibit (i.e., ROCK 1, ROCK 2, or both). However, evidence from a previous study revealed that ROCK 2 is a regulator of apoptosis, axonal degeneration, and neurite outgrowth in the central nervous system (CNS) and that ROCK 2 downregulation increases RGC survival in a rat NC model [38]. Thus, the specific mechanism by which Y-27632 promotes neuroprotection may be related to ROCK 2.

In addition, some therapeutic strategies involving a series of mechanisms, such as modulating neurotrophic factors, oxidative stress, the calcium channel pathway, nitric oxide levels, heat shock protein expression, immunology, and neuroprotection, may slow or even reverse RGC apoptosis [3]. The results of the present study also demonstrated that Y-27632 has non-IOP-related neuroprotective effects and provided additional evidence. Since RGCs participate in cross-talk with other nerve cells and glial cells in the retina, the effects of Y-27632 may occur through these cells. To address the limitations of the influence of other cells, a pure isolated RGC culture system was selected for our in vitro study, even though isolated RGCs have limited viability. In the present study, at the highest concentration of Y-27632, the RGC survival rate remained high, but the dead/live cell ratio increased. This finding may imply that this ROCK inhibitor induces complex cellular responses, which may include cellular stress, a shift in the mode of cell death, changes in cell cycle progression, or nonspecific toxicity. While many cells survive initially, the increase in the dead/live RGC ratio points to the possibility that the drug may cause delayed cell death or irreversible cellular damage. However, the detailed mechanism of Y-27632-related neuroprotection of RGCs still lacks direct evidence. Thus, further studies are needed to clarify the specific mechanism underlying these observations.

According to previous reports, the NC model is similar to glaucomatous injury, which can induce RGC death and represents an acute approach to rapidly establish optic nerve damage models [27,39]. Two common in vivo experimental models of glaucoma are the mechanical damage model and the vascular damage model. Several animal models of glaucoma have been proposed; however, they present various challenges, including inconsistent success rates in establishing models with elevated IOP, difficulty maintaining a stable IOP, and the unintended induction of inflammation, which can lead to greater variability in outcomes. Since this study aimed to investigate the neuroprotective effect of Y-27632 administered via eye drops, its effects were considered potentially weaker than those of oral or intraperitoneal administration. Therefore, the NC model was employed in the current study to minimize measurement variability as much as possible. However, glaucoma is a chronic optic neuropathy characterized by the progressive degeneration of RGCs, which demonstrates the limitations of the NC model. Thus, confirming the effects of Y-27632 in other glaucoma models is necessary. The effect of Y-27632 on retinal thickness, as examined by OCT in this study, was not statistically significant overall. However, a slight trend toward improvement was observed in the inner layers of certain areas, although this was not consistent with the findings from retinal whole-mount specimens and ERG waveforms. The difference in detection sensitivity between examination methods may be a contributing factor, but further studies are needed to clarify this.

The present study suggested that Y-27632 delayed RGC apoptosis in a concentration-dependent manner. Although the highest concentration tested was the most effective, further investigation is needed to assess the effects of Y-27632 across a broader range of concentrations. Currently, two types of ROCK inhibitors have been approved for clinical use; however, their direct neuroprotective effects remain to be fully elucidated in future studies. While only a single concentration was evaluated in vivo, in vitro experiments revealed a dose-dependent neuroprotective effect at specific concentrations. Notably, although the 1000 nM Y-27632 exhibited neuroprotective activity, the high variability observed at this concentration suggests that lower doses may provide more consistent efficacy. These findings underscore the need for additional in vivo studies to determine the optimal therapeutic dose of ROCK inhibitors. While the present study provides evidence supporting the direct neuroprotective effect of Y-27632 on RGCs, further research is warranted to explore other potential mechanisms contributing to this effect. Moreover, as this study employed a mouse model, validation in other animal models with greater physiological similarity to humans is essential to evaluate the effectiveness and safety of Y-27632.

In summary, our results provide compelling evidence that ROCK inhibitors, such as Y-27632, can reduce IOP and may also be promising medicines for certain optic neuropathies, such as traumatic optic neuropathy and glaucoma.

## Data Availability

The raw data supporting the conclusions of this article are available from the corresponding author upon reasonable request.
